# Drivers of house invasion by sylvatic Chagas disease vectors in the Amazon-Cerrado transition: A multi-year, state-wide assessment of municipality-aggregated surveillance data

**DOI:** 10.1371/journal.pntd.0006035

**Published:** 2017-11-16

**Authors:** Raíssa N. Brito, David E. Gorla, Liléia Diotaiuti, Anália C. F. Gomes, Rita C. M. Souza, Fernando Abad-Franch

**Affiliations:** 1 Grupo Triatomíneos, Instituto René Rachou–Fiocruz Minas Gerais, Belo Horizonte, Minas Gerais, Brazil; 2 Laboratorio de Eco-Epidemiología Espacial de Enfermedades Transmitidas por Vectores, Instituto de Altos Estudios Espaciales Mario Gulich–CONAE / Universidad Nacional de Córdoba–CONICET, Falda del Cañete, Córdoba, Argentina; 3 Coordenação de Vigilância de Doenças Vetoriais e Zoonoses, Secretaria Estadual de Saúde do Tocantins, Palmas, Tocantins, Brazil; Tulane University School of Public Health and Tropical Medicine, UNITED STATES

## Abstract

**Background:**

Insecticide spraying efficiently controls house infestation by triatomine bugs, the vectors of *Trypanosoma cruzi*. The strategy, however, is ineffective against sylvatic triatomines, which can transmit Chagas disease by invading (without colonizing) man-made structures. Despite growing awareness of the relevance of these transmission dynamics, the drivers of house invasion by sylvatic triatomines remain poorly understood.

**Methods/Findings:**

About 12,000 sylvatic triatomines were caught during routine surveillance in houses of Tocantins state, Brazil, in 2005–2013. Using negative binomial regression, information-theoretic model evaluation/averaging, and external model validation, we investigated the effects of regional (Amazon/Cerrado), landscape (preservation/disturbance), and climate covariates (temperature, rainfall) on the municipality-aggregated numbers of house-invading *Rhodnius pictipes*, *R*. *robustus*, *R*. *neglectus*, and *Panstrongylus geniculatus*. House invasion by *R*. *pictipes* and *R*. *robustus* was overall more frequent in the Amazon biome, tended to increase in municipalities with more well-preserved land, and decreased in rainier municipalities. Across species, invasion decreased with higher landscape-disturbance levels and in hotter-day municipalities. Invasion by *R*. *neglectus* and *P*. *geniculatus* increased somewhat with more land at intermediate disturbance and peaked in average-rainfall municipalities. Temperature effects were more pronounced on *P*. *geniculatus* than on *Rhodnius* spp.

**Conclusions:**

We report widespread, frequent house invasion by sylvatic triatomines in the Amazon–Cerrado transition. Our analyses indicate that readily available environmental metrics may help predict the risk of contact between sylvatic triatomines and humans at coarse geographic scales, and hint at specific hypotheses about climate and deforestation effects on those vectors–with some taxon-specific responses and some seemingly general trends. Thus, our focal species appear to be quite sensitive to higher temperatures, and might be less common in more heavily-disturbed than in better-preserved environments. This study illustrates, in sum, how entomological routine-surveillance data can be efficiently used for Chagas disease risk prediction and stratification when house-colonizing vectors are absent.

## Author summary

Triatomine bugs are the vectors of Chagas disease, still a key public health concern in the Americas. Insecticide spraying efficiently controls house infestation by triatomines, but is useless against sylvatic bugs–which can transmit the disease by simply invading human residences. Although this behavior is common, the drivers of house invasion by wild triatomines remain poorly understood. Using municipality-aggregated data from routine surveillance, we investigated whether and how some major environmental factors affect house invasion by four triatomine species across the transition between Amazon rainforests and Cerrado savannahs in Brazil. We found that house invasion (i) is widespread, (ii) varies by region for some species, (iii) is overall less frequent in areas with higher levels of landscape disturbance, and (iv) is less common in hotter and in rainier sites. Although the effects of landscape disturbance and climate differed somewhat among bug species, the general approach we describe here may help advance Chagas disease risk assessment when house-colonizing vectors are absent.

## Introduction

Chagas disease is caused by infection with *Trypanosoma cruzi*, a protozoan parasite transmitted by blood-sucking bugs known as triatomines [1]. The disease is endemic across Latin America, where about 5–6 million people are thought to be infected and over 70 million live at risk of contagion [2]. The prevalence of human infection is typically higher in areas where triatomines colonize (i.e., breed) in houses or peridomestic structures [3,4]. Indoor residual-insecticide spraying efficiently reduces house infestation and colonization, yet re-infestation often ensues a few weeks or months after spraying [4,5]. Re-infestation, in turn, usually starts when adult triatomines invade houses by flying from nearby infestation foci located in either natural or man-made ecotopes [5–7]. Re-spraying houses every time one invading bug is found indoors would clearly be unsustainable–it would not affect the source bug populations, would over-expose residents to harmful chemicals, and would increase the odds of selecting insecticide-resistant vectors.

House invasion by adult triatomines has been recorded in rural, urban, and suburban settings across tropical and subtropical America [8–15]. Since, once infected with *T*. *cruzi*, triatomines usually retain infection for life, house-invading adult (i.e., winged) bugs often carry the parasite. Domestic *T*. *cruzi* transmission is thus possible even in the absence of house colonization; it can occur either through direct vector-human contact (i.e., classical stercorarian transmission) [16,17] or through the contamination of food or beverages by infected vectors, which may lead to oral transmission [18]. This latter mechanism has been implicated in outbreaks of acute, often severe Chagas disease reported (with increasing frequency since about 2005) from across South America [3,19–21].

Despite growing awareness of the relevance of *T*. *cruzi* transmission by house-invading triatomines, we know rather little about how frequently these vectors enter houses; furthermore, the environmental drivers of such invasive behavior remain largely uncharted (but see [10,13]). We examined these issues using a multi-year, state-wide, municipality-aggregated triatomine house-invasion dataset from the Amazon–Cerrado transition in Tocantins, Brazil. We focused on four common species that seldom colonize in houses–two typical of the Amazon moist forests (*Rhodnius pictipes* and *R*. *robustus s*.*l*.), one typical of the seasonally dry Cerrado savannahs (*R*. *neglectus*, which can breed in man-made structures but hardly ever does so in our study region), and one recorded across most of Latin America (*Panstrongylus geniculatus*). We specified *a priori* alternative hypotheses (outlined in Table 1) about the possible effects of regional, landscape, and climate covariates on the numbers of house-invading bugs of each species. We then derived a set of predictions under each hypothesis–what, given our knowledge about the bugs’ biology, would be the expected effect of each covariate on the numbers of house-invading vectors (see Table 1). Using a multi-model inference approach, we found that widely available environmental metrics may help predict house invasion by sylvatic triatomines at coarse geographic scales; further, landscape-disturbance and climate effects differed among bug species, suggesting taxon-specific responses to common sources of environmental stress.

**Table 1:** Main *a priori* hypotheses (and predictions) about the effects of environmental covariates on the numbers of house-invasion events by sylvatic triatomines, with examples of related negative binomial (count) model structures.

Category	Hypothesis and predictions	Count model structure
Null	House invasion by sylvatic triatomines varies randomly across municipalities	Y(.)
	House invasion depends on the number of inhabited houses (considered ‘available’ for invasion) in each municipality, but may also independently increase with worse average housing conditions (with the Human Development Index [*HDI*] used as a proxy)	Y(House*+Human Development Index*)
Regional	The number of house invasion events varies depending on the extent of municipal territory in different biomes–with more invasion events by the typically Amazonian *Rhodnius robustus* or *R*. *pictipes*, and less invasion events by the Cerrado-associated *R*. *neglectus*, in municipalities with more land within the Amazon biome; no effect is expected for *Panstrongylus geniculatus*	Y(*H*+*HDI*+*Amazon*)
Landscape	House invasion depends primarily on landscape disturbance levels, with less invasion events in municipalities with more well-preserved land, where more complex food-webs provide a tighter control of bug population growth	Y(*H*+*HDI*+*Preserved*)
	House invasion depends primarily on landscape disturbance levels, with overall less invasion events in municipalities with more heavily-disturbed land, where the loss of suitable habitat (and perhaps hosts) limits bug population growth	Y(*H*+*HDI*+*Disturbed*)
	House invasion depends on the degree of landscape disturbance, with more invasion events in municipalities with more land at intermediate disturbance levels–with simplified food-webs and fair habitat/host availability	Y(*H*+*HDI*+*Intermediate*)
	House invasion depends on landscape features summarized in the NDVI ‘greenness’ metric, with positive effects on the moist forest-dwelling *R*. *pictipes* and *R*. *robustus* and negative effects on the savannah-dwelling *R*. *neglectus*	Y(*H*+*HDI*+*NDVI*)
Climate	Climate drives house invasion primarily through high diurnal temperatures, which limit bug survival and population growth and hence result in an overall reduction of invasion events in hotter-day municipalities	Y(*H*+*HDI*+*Day*)
	Climate drives house invasion primarily through nocturnal temperatures, which, when low, may inhibit flight initiation by the bugs–and hence result in an overall increase of invasion events in warmer-night municipalities	Y(*H*+*HDI*+*Night*)
	Climate drives house invasion primarily through temperature amplitude, with larger *ΔT* values combining the negative effects of high diurnal and low nocturnal temperatures–and hence resulting in less invasion events	Y(*H*+*HDI*+*ΔT*)
	Climate drives house invasion events simply because heavy rainfall physically hampers bug flight; this results in less invasion events in rainier municipalities (which, in Tocantins, means areas with very heavy seasonal rains)	Y(*H*+*HDI*+*Rain*)
Joint	House invasion by triatomine species typical of either the Amazon or the Cerrado varies across biomes (as above for *Amazon*) and also, independently, across landscape disturbance levels (as above for *Preserved*)	Y(*H*+*HDI*+*Amazon*+*Preserved*)
	House invasion by Amazon/Cerrado sylvatic triatomines is independently affected by regional differences (as above for *Amazon*) and higher day temperatures (as above for *Day*)	Y(*H*+*HDI*+*Amazon*+*Day*)
	House invasion by Amazon/Cerrado sylvatic triatomines is independently affected by regional variation (as above for *Amazon*), landscape disturbance levels (as above for *Preserved*), and higher day temperatures (as above for *Day*)	Y(*H*+*HDI*+*Amazon*+*Preserved*+*Day*)
	House invasion by Amazon/Cerrado sylvatic triatomines is independently affected by regional variation (as above for *Amazon*), landscape disturbance levels (as above for *Preserved*), higher day temperatures (as above for *Day*), and heavier rainfall (as above for *Rain*)	Y(*H*+*HDI*+*Amazon*+*Preserved*+*Day*+*Rain*)

Y, dependent variable (number of bugs caught invading houses); Y(.) represents the intercept-only model

*House (abbreviated *H*) and the *Human Development Index* (*HDI*) were considered as potential confounders and included in regional, landscape, and joint models–where the estimated effects of covariates are therefore independent of the number of houses and of the Human Development Index value (a proxy for housing conditions) in each municipality

See the main text for a detailed definition of each regional, landscape, and climate covariate

## Methods

### Setting

**Fig 1:**
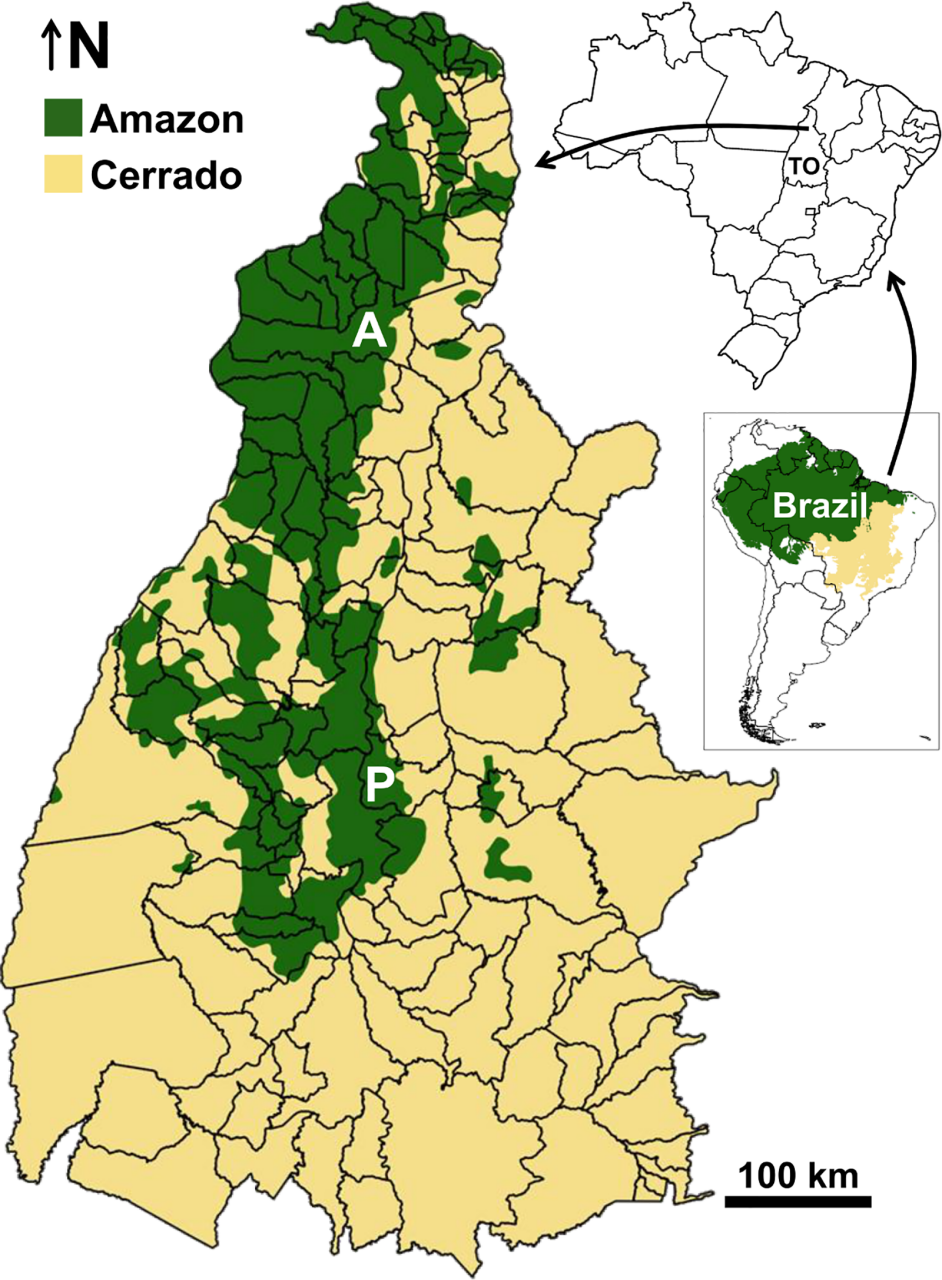
The state of Tocantins (TO), Brazil. The map shows municipality limits (with **P** and **A** highlighting the two largest urban centers, Palmas and Araguaína) and the approximate location of the boundary between the Amazon and Cerrado biomes. Biome boundaries were drawn using shapefiles available from The Nature Conservancy at http://maps.tnc.org/gis_data.html#TerrEcos.

Tocantins state, Brazil, comprises 139 municipalities covering ~280,000 km^2^ (Fig 1); about 1.5 million people live in the state [22]. The two largest urban centers are Palmas (~270,000 inhabitants) and Araguaína (~170,000 people) [22]. Most of Tocantins lies in the seasonally dry Cerrado (the savannah of central Brazil), but to the northwest it covers part of the transition between the Cerrado and the moister southeastern Amazon; a complex forest/savannah mosaic extends along that transition (Fig 1). At least 16 triatomine species occur naturally in Tocantins, including species that colonize in man-made structures and species that may invade houses or other premises but rarely, if ever, breed there (Table 2). Reliable data on the epidemiology of *T*. *cruzi* infection in Tocantins are wanting [23,24]. In 2007–2013, the Ministry of Health recorded 23 cases of acute Chagas disease (all age classes) and 350 deaths (all >30 year-olds) caused by the disease in Tocantins–a datum overall consistent with mortality analyses for 1999–2007 [25–27]. In 1992, Tocantins blood banks detected *T*. *cruzi* infection in 0.75% of donors [28]. Sporadic reports describe isolated cases or small outbreaks likely related to oral transmission [29–31], and Vinhaes et al. [32] classified 14% of Tocantins municipalities as ‘highly vulnerable’ to vector-borne Chagas disease. No *T*. *cruzi* infections were detected, however, in 1168 children ≤ 5 years old sampled between 2001 and 2008 [33]. Overall, these fragmentary data suggest low-intensity, yet continuous, transmission of the parasite to people in Tocantins. Sylvatic transmission cycles involving wild triatomines and mammals are probably widespread in the state, and infection of domestic dogs can be locally common [34,35]. Importantly, most new cases of human Chagas disease in Tocantins are reported from northern municipalities [25,29–31], where triatomines seldom colonize in man-made structures; these cases are therefore most likely linked to sylvatic vectors that invade houses and transmit the parasite either directly [16,17] or by contaminating food or beverages [18–21].

**Table 2:** Triatomine bugs caught inside or around dwellings in the state of Tocantins, Brazil (2005–2013), and their infection with *Trypanosoma cruzi*.

Species (ever reported)	Colonization	Captured	Nymphs	Examined (OM)	% infected (CI)
***Rhodnius pictipes***	Very rarely	4624	37	4593	25.6 (24.4–26.9)
***Rhodnius robustus***	Not reported	783 (653)*	2 (0)*	783	32.3 (29.1–35.7)
***Rhodnius neglectus***	In some areas	2433 (1118)*	93 (7)*	2383	13.1 (11.8–14.5)
***Panstrongylus geniculatus***	Very rarely	2889	22	2883	10.8 (9.7–12.0)
*Triatoma sordida*	Common	18,395	9584	18,249	1.7 (1.5–1.9)
*Triatoma costalimai*	Rarely	816	365	762	13.5 (11.3–16.1)
*Triatoma pseudomaculata*	Common	546	89	544	5.5 (3.9–7.8)
*Panstrongylus diasi*	Occasionally	115	0	113	1.8 (0.5–6.2)
*Panstrongylus lignarius*	Not reported	101	0	98	29.6 (21.5–39.3)
*Panstrongylus megistus*	In some areas	45	13	38	0.0 (0.0–9.2)
*Eratyrus mucronatus*	Very rarely	11	0	11	0.0 (0.0–25.9)
*Triatoma brasiliensis*	Common	9	0	9	11.1 (1.98–43.5)
*Cavernicola pilosa*	Extremely rarely	3	0	3	0.0 (0.0–56.2)
*Triatoma jatai*	Not reported	3	0	3	0.0 (0.0–56.2)
*Psammolestes tertius*	Not reported	0	0	-	-
*Microtriatoma trinidadensis*	Not reported	0	0	-	-

The four focal species included in our main analyses are in **bold** typeface

“Colonization” refers to the ability to colonize (i.e., establish self-sustaining breeding colonies) in man-made structures; note that these are overall trends that disregard many details about the behavior of local populations (for example, a *Panstrongylus lignarius*-like population known as *P*. *herreri* is often found infesting houses in the Andean stretches of the Marañón river valley of northeastern Peru, and there is a great deal of variation in this trait among members of the *Triatoma brasiliensis* species complex)

“Examined” refers to the number of bugs examined by optical microscopy (OM) for infection with *Trypanosoma cruzi* (see Methods); percentages of bugs found infected are presented with score 95% confidence intervals (CIs)

*Tocantins vector surveillance staff was trained to distinguish *Rhodnius neglectus* and *R*. *robustus*, two near-sibling species, in 2009; we therefore used only the data for 2010–2013 (here in parentheses) in our main analyses

### The data

Our data on house-invading sylvatic triatomines come from the entomological surveillance system coordinated by Tocantins state health department. This decentralized system works primarily at the municipality level and combines two sources of information about triatomines. First, dwellers can notify the presence of suspect insects in their homes to health agents; second, health agents actively search for triatomines in and around houses in pre-determined schedules [36]. Triatomines are initially identified and checked for *T*. *cruzi* infection (through optical microscopy of hindgut contents) by trained staff in local laboratories. All insects (including those identified as non-triatomines) and all microscope slides (irrespective of the results of the first examination) are then sent to the state health department headquarters for quality control [36]. When triatomines are found in a dwelling, health agents take action as prescribed by technical guidelines–which include, depending on the situation, residual insecticide spraying, dwelling-level environmental management, education, and/or testing residents for *T*. *cruzi* infection [36].

For the present analyses, we selected the four sylvatic triatomine species most commonly reported invading houses in Tocantins (Table 2). *Rhodnius pictipes* and *R*. *robustus* occur throughout the Amazon-Orinoco lowlands, whereas *R*. *neglectus* is primarily associated with the Cerrado savannahs and gallery forests [37–39]. These *Rhodnius* species are all typical palm dwellers [40] and occur in sympatry in Tocantins [9,39]. *Panstrongylus geniculatus* has been recorded from Mexico to Argentina and at sites ranging from rainforests to deserts [1,37,41]. For the easy-to-identify *R*. *pictipes* and *P*. *geniculatus* [1] we analyze house-invasion data for the period 2005–2013; for *R*. *robustus* and *R*. *neglectus*, which have very similar phenotypes [1,9], we restrict our analyses to records produced after 2009 (i.e., the period 2010–2013), when vector surveillance staff across Tocantins received specific training to distinguish these two species. We finally used data for the period 2014–2016 as an independent check of model predictions (see below). Most entries in the database included the name of the site where each vector was found; however, the coordinates of those sites were not recorded and, for many of them (e.g., farms or small rural communities), were unavailable in gazetteers. We therefore base our (largely exploratory) analyses on the municipality-aggregated numbers of specimens found invading houses across Tocantins state over the time-periods specified above. The full raw data are provided as supporting information (S1 Data).

### Covariates and confounders

We explored the effects of three major sets of environmental descriptors, which were included as covariates in generalized linear models (GLMs, see Table 1 and below). We first specified a *regional-scale* covariate to investigate biome-level differences in the frequency of house invasion by each triatomine species (Table 1). We used biome (The Nature Conservancy; http://maps.tnc.org/gis_data.html#TerrEcos) and municipality (Brazilian Institute of Geography and Statistics, IBGE; www.ibge.gov.br) digital shapefiles to calculate, for each municipality and using Quantum GIS 2.14.3 (QGIS; http://www.qgis.org/) and GRASS 7.0.4 (https://grass.osgeo.org/), the percent of territory within the Amazon biome (covariate ‘*Amazon*’)–with the rest corresponding to Cerrado savannahs (Fig 1). Second, we explored possible *landscape-scale* effects (Table 1) by roughly measuring the degree of landscape preservation/disturbance based on IBGE land-use data published in 2010 (see ftp://geoftp.ibge.gov.br/informacoes_ambientais/cobertura_e_uso_da_terra/uso_atual/mapas/brasil/uso_da_terra_2010.pdf). The IBGE divides municipalities into census tracts and assigns each tract to one of five land-use classes including built environments (mainly towns, roads, and industrial complexes) and rural areas in which farmlands occupy either >50%, 50–25%, 24.9–10%, or <10% of the surface–with the remaining percentage corresponding to preserved forest or savannah. Using this classification and QGIS, we calculated the percent of territory (excluding water bodies) in each municipality corresponding to three coarse categories: (i) preserved land with <25% occupied for farming (covariate ‘*Preserved*’), (ii) disturbed land, including built environments and mixed areas with >50% of land occupied for farming (covariate ‘*Disturbed*’), and (iii) land under intermediate disturbance, i.e., mixed areas with between 25% and 50% of land occupied for farming (covariate ‘*Intermediate*’). We also assessed the potential utility of a single-number landscape metric, the Normalized Difference Vegetation Index (covariate ‘*NDVI*’) derived from the MODerate-resolution Imaging Spectroradiometer (MODIS; data for 2005–2013, 250-m spatial resolution and 16-day temporal resolution; http://glovis.usgs.gov/) [42]. The *NDVI* covariate quantifies mean photosynthetic activity (“greenness”) in each municipality [42]. Although NDVI values may saturate in dense-canopy forest areas [42], limiting our ability to distinguish Amazon rainforests from some Cerrado dense forests, the metric would still be useful for assessing rough landscape-scale differences in average vegetation land-cover among municipalities. We finally investigated possible effects of *climate* (Table 1) covariates including (i) mean diurnal (covariate ‘*Day*’) and mean nocturnal (covariate ‘*Night*’) land surface temperatures (MODIS; 2005–2013, 1-km and 8-day resolution), plus temperature amplitude (covariate ‘*ΔT*’ = *Day*–*Night*); and (ii) mean annual rainfall (covariate ‘*Rain*’), with data from the Tropical Rainfall Measuring Mission (TRMM; 2005–2013, 25-km and 30-day resolution; http://trmm.gsfc.nasa.gov/). Satellite images were read, format-transformed, and re-projected to the WGS84 frame using the MODIS Reprojection Tool (https://lpdaac.usgs.gov/tools/modis_reprojection_tool), then re-sampled to 250-m spatial resolution using R package rgeos; the values of interest were averaged for each municipality using packages rgdal, raster, and maptools in R 3.1.2 [43–47]. We note that some of our covariates provide complementary descriptions of the same phenomena; for example, *ΔT* is a function of diurnal and nocturnal temperatures. We avoided including redundant covariates together in any single model. We also note that we did not test for interactions among covariates because we could not specify *a priori* how the effects of any covariate would vary across values of any of the other covariates at the coarse scale of our analyses. For example, we did not see why or how the effects of municipality-averaged temperatures would differ at varying levels of landscape disturbance or between two adjacent biomes assessed along their contact zone.

We finally considered three *potential confounders* in our analyses (Table 1). First, we controlled for the log-transformed number of inhabited houses (i.e., those considered ‘available’ for invasion by triatomines) in each municipality (‘*House*’, with data from the 2010 IBGE census). Second, we thought that worse housing conditions in municipalities with lower levels of socioeconomic development or more widespread poverty might result in more house-invasion events. We therefore evaluated the use of two variables to coarsely adjust for the possible effects of municipality-specific values of (i) the Human Development Index [48] (‘*HDI*’, with data from the 2010 IBGE census), and (ii) the percent of people classified as poor by the IBGE (‘*Poverty*’, with data from the 2002–2003 IBGE household budget survey). We separately developed full sets of *HDI*- and *Poverty*-adjusted models and compared their fit to the data using the small-sample version of Akaike’s information criterion (AICc; see ref. [49] and below); we will focus here on the results of the overall better-performing *HDI*-adjusted models, but will also comment on *Poverty*-adjusted models in the **Results** and **Discussion** sections. Summary statistics of covariates and confounders are provided in S1 Table and mapped values in S1 Fig.

### Data analyses

We first summarized data on municipality-level house-invading triatomines and environmental covariates in tables and graphs, and conducted exploratory bivariate analyses with covariates standardized to mean zero and standard deviation one. We then used generalized linear models (GLMs) to investigate the relationships between the numbers of house-invading triatomines and our set of environmental covariates across Tocantins municipalities. For each species, we pre-specified a set of GLMs representing specific versions of the general *a priori* hypotheses outlined in Table 1 and below. We fitted GLMs via maximum likelihood and evaluated their relative performance with AICc [49]. Using AICc, we first checked what error distribution would best fit our count data; for all species and model specifications, the negative binomial distribution was preferred over the Poisson distribution. Because *R*. *pictipes* and *R*. *robustus* are typically Amazonian [37,38], we considered it unlikely that they would occur in all of Tocantins municipalities–many of which are dominated by Cerrado vegetation (Fig 1). In other words, our *R*. *pictipes* and *R*. *robustus* data likely contain two kinds of zeros: true or structural zeros in municipalities where these species do not occur, and false or observation zeros in municipalities where they do occur but were never recorded by the vector surveillance system [50]. Given the mosaic nature of the Amazon–Cerrado transition in Tocantins (Fig 1), it was however unclear which municipalities might actually be out of the range of these two species. To account for the two potential sources of zeros, we analyzed *R*. *pictipes* and *R*. *robustus* data using zero-inflated negative binomial (ZINB) GLMs [50,51]. ZINB models use a mixture of a point mass at zero for modeling the excess zeros (with covariates on presence/absence evaluated using the logit link function) and a negative binomial distribution for modeling the counts (with covariates evaluated using the log link function) [50,51]. On the other hand, we did not expect any true zeros in the *P*. *geniculatus* or *R*. *neglectus* data–the former occurs across Latin America and the latter along the southeastern Amazon fringe including northwestern Tocantins [9,37–41]. For these two species, therefore, we used standard negative binomial (NB) GLMs [51]. Our count models fall into five major categories (Table 1 and S2 Table).

1. Null models representing the null hypothesis of random variation of house invasion by sylvatic triatomines across municipalities; we considered models estimating only intercepts and models including only potential confounders.
2. Regional-scale models representing the general hypothesis that invasion by *R*. *pictipes* and *R*. *robustus* should be more frequent, and invasion by *R*. *neglectus* perhaps less so, in municipalities with more territory within the Amazon biome [1,9,37–40]; we did not expect any regional effect for the wide-ranging *P*. *geniculatus* [1,37,39,41].
3. Landscape-scale models representing the general hypothesis that house invasion by sylvatic triatomines should be less frequent in areas with either more heavily-disturbed land (where triatomine populations should be rarer if habitat loss leads to local extinction) or more well-preserved landscapes (where triatomine populations may be closer to equilibrium with their hosts, predators, and pathogens, resulting in less dense colonies and reduced dispersal) [13,15,34,52,53]; we also considered alternative model specifications testing the effects of intermediate levels of disturbance [13,15] and of *NDVI* [39] as a single-number landscape-scale covariate (Table 1).
4. Climate models representing the general hypothesis that physiological constraints imposed on triatomines by temperature extremes should result in overall less frequent invasion in municipalities with (i) higher day temperatures (which can limit triatomine survival), (ii) lower night temperatures (which may inhibit flight initiation), and, hence, (iii) larger temperature ranges [1,54–58]; heavy rainfall, on the other hand, could physically hamper dispersive flight by triatomines [57,58]. After exploratory analyses, we also tested for non-linear effects of rainfall on *R*. *neglectus* and *P*. *geniculatus* using a quadratic *Rain* term (see S2 Table).
5. Joint models containing different combinations (guided by the results of interim analyses within model categories 2 to 4) of regional-scale, landscape-scale and climate covariates; we note that the binomial part of our ZINB models also included different combinations of climate and regional covariates (see all models in S2 Table).

Models were fitted using packages MASS and pscl in R 3.1.2 [43,59–61]. For each species, covariate effect-size estimates and predictions were averaged across models using Akaike weights [49] in AICcmodavg [62]. We base inference on the full results of each model set’s analysis, and specifically on (i) the covariate structure of better- *vs*. worse-performing models, as assessed by AICc (with lower scores signaling a better compromise between model fit and model complexity), and (ii) the sign, size, and unconditional standard errors of model-averaged, adjusted effect estimates for each covariate [49]. Each species’ full model set and model AICc ranks are provided in S2 Table. As mentioned above, we used data collected in the years 2014, 2015, and 2016, which were not available as we were developing our focal analyses, to test the predictions of each species’ models. For this external validation [63], we compared the numbers of house-invading bugs predicted by the models (on a per-year basis) with those actually recorded in 2014–2016 using (i) Pearson’s product moment correlation coefficient (*ρ*); (ii) mean bias error (MBE = Σ(*Ŷ*_*i*_−*Y*_*i*_) / *N*); and (iii) mean absolute error (MAE = Σ(|*Ŷ*_*i*_−*Y*_*i*_|) / *N*). Here, Σ(*Ŷ*_*i*_−*Y*_*i*_) is the sum over municipalities (for *i* = 1 to 139) of the differences between (per-year) model-predicted values (*Ŷ*_*i*_) and observed 2014–2016 values (*Y*_*i*_); |*Ŷ*_*i*_−*Y*_*i*_| is the absolute value of each difference; and *N* = 139 is sample size [63].

## Results

### General descriptive results

Table 2 shows the number of triatomines captured inside or around dwellings of Tocantins over the study period. Of the 16 species previously recorded in the state, only *Psammolestes tertius* and *Microtriatoma trinidadensis* were not represented in this sample (Table 2). Of central interest to our investigation were the large numbers of house-invading bugs of species that very rarely, if ever, colonize in man-made structures. In particular, 10,729 specimens of *R*. *pictipes*, *R*. *robustus*, *R*. *neglectus*, and *P*. *geniculatus* were caught in human dwellings from 2005 to 2013. Excluding *R*. *robustus* and *R*. *neglectus* data from 2005–2009 (see Methods), we analyzed 9284 records of these four focal species (Table 2 and S1 Data).

### Rhodnius pictipes

**Fig 2:**
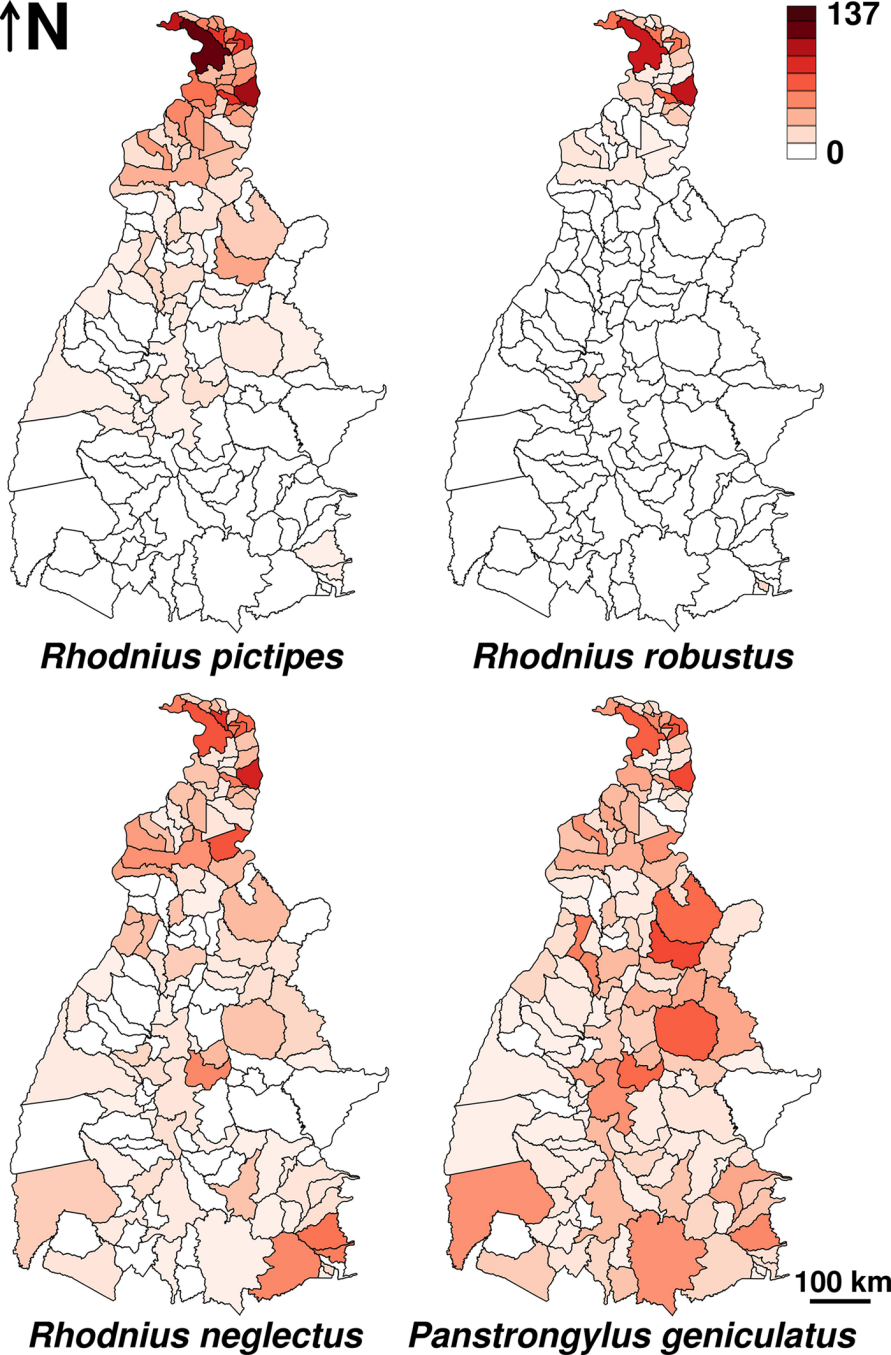
Observed house invasion events (on a per-year basis) by four sylvatic triatomine species in Tocantins, Brazil. The maps show the limits of the 139 municipalities in the state of Tocantins. Darker shades of red indicate more invasion events; raw values were transformed to log_10_(*y*+1) to improve resolution at the lower end of the range.

Among the four focal sylvatic species, *R*. *pictipes* was the most frequently found invading houses in Tocantins. Sixty-six municipalities reported house invasion by *R*. *pictipes*, with most bugs found indoors (95.9% of 4624) and often infected with *T*. *cruzi* (Table 2, Fig 2). *R*. *pictipes* catches were recorded year-round, with a peak in May-June; of the 4557 house-invading specimens that were sexed, 53.7% (score 95% confidence interval [CI], 52.3–55.1%) were females.

Table 3 presents the subset of *R*. *pictipes* ZINB models with ΔAICc < 2.0. These six best-fitting GLMs were all ‘joint models’ including regional-scale (*Amazon*), climate (*Rain* plus *Day* or *ΔT*), and, except for two models, landscape-scale (*Preserved*, *Disturbed*) covariates in the negative binomial (count) sub-model. Null models, as well as regional-scale, landscape-scale, and climate-only models, all performed substantially worse than the joint models shown in Table 3 (see S2 Table).

**Table 3:** Covariate structure of the top-ranking count models (ΔAICc < 2.0) within each triatomine species’ model set.

Species	Model	ΔAICc	Regional	Landscape				Climate			
			*Amazon*	*Preserved*	*Intermediate*	*Disturbed*	*NDVI*	*Day*	*Night*	*ΔT*	*Rain*
*Rhodnius pictipes*	ZINB pict28	0	●	●				●			●
	ZINB pict21	0.668	●					●			●
	ZINB pict30	0.715	●	●						●	●
	ZINB pict35	0.889	●			●		●			●
	ZINB pict23	1.290	●							●	●
	ZINB pict37	1.760	●			●				●	●
*Rhodnius robustus*	ZINB rob11	0						●			●
	ZINB rob56ndvi	0.341					●	●			●
	ZINB rob56	0.392				●		●			●
	ZINB rob35ndvi	0.633	●				●	●			●
	ZINB rob11|*Day*	1.038						●			●
	ZINB rob52	1.049				●		●			
	ZINB rob56interm	1.490			●			●			●
	ZINB rob56|*Day*	1.551				●		●			●
	ZINB rob27	1.620	●	●							●
	ZINB rob21	1.630	●					●			●
	ZINB rob49	1.696		●				●			●
	ZINB rob35	1.707	●			●		●			●
*Rhodnius neglectus*	NB neg78ndvi	0					●	●			●^2^
	NB neg78	1.488			●			●			●^2^
	NB neg64	1.787				●		●			●^2^
	NB neg50	1.905	●		●			●			●^2^
*Panstrongylus geniculatus*	NB genic60	0				●		●			
	NB genic64	0.482				●		●			●^2^
	NB genic62	0.947				●				●	
	NB genic66	1.879				●				●	●^2^

● Covariate included in the model (with ‘|*Day*’ indicating alternative binomial sub-models; see S2 Table); ●^2^ indicates that the model includes a quadratic *Rain* term

AICc, Akaike’s information criterion corrected for finite sample size (ΔAICc, difference from the top-ranking model); note that all models included also two potential confounders–for each municipality, the number of *Houses* and the Human Development Index (*HDI*)

**Fig 3:**
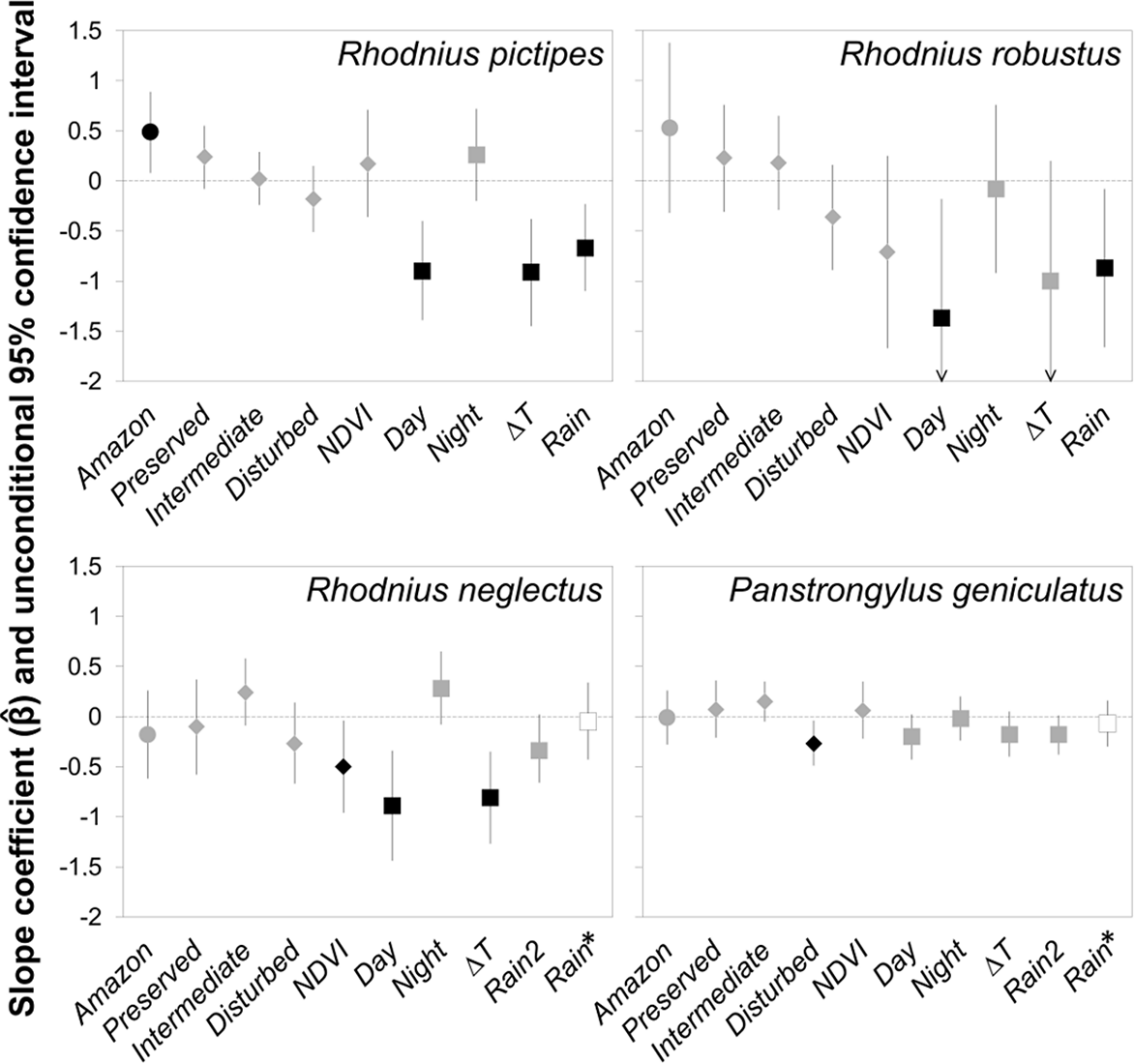
Model-averaged estimates of covariate effects on house invasion by four sylvatic triatomine species. Circles, regional-scale covariate (*Amazon*); diamonds, landscape-scale covariates (*Preserved*, *Intermediate*, *Disturbed*, *NDVI*); squares, climate covariates (*Day*, *Night*, *ΔT*, *Rain*; *Rain*2, quadratic *Rain* term; Rain*, estimate and confidence interval (CI) from the top-ranking model). Effects are considered different from zero (black symbols) when the 95% CIs do not cross the horizontal line at zero. (Arrowheads on the CI bars for *Day* and *ΔT* in the *Rhodnius robustus* panel indicate that the lower CI limits are out of the graphed range.) See Table 3 and S1–S6 Tables for covariates, model sets, and the values of effect estimates and CI limits; model-averaged estimates from *Poverty*-adjusted model sets are presented in S2 Fig.

In line with the regional hypothesis, the model-averaged *Amazon* coefficient predicted more house-invasion events in municipalities with more land within Amazonia (Fig 3); this effect was slightly attenuated in *Poverty*-adjusted models (S2 Fig). Invasion by *R*. *pictipes* appeared to become somewhat more common in municipalities with more well-preserved land and somewhat rarer in those with more heavily-disturbed landscapes, although the CIs included zero (Fig 3, S2 Fig). We found no evidence for any effects of land at intermediate disturbance or of NDVI on house invasion by *R*. *pictipes*–first, these covariates were not present in our best-fitting models (Table 3), and, second, their coefficients were very close to zero (Fig 3, S3 Table; see also S2 Fig). We finally estimated negative effects of higher diurnal temperatures, larger temperature amplitudes, and rainfall (Fig 3, S3 Table S2 Fig). The point estimate of the effect of higher night temperatures was positive; the CI included zero in *HDI*-adjusted but not in *Poverty*-adjusted models (Fig 3, S3 Table, S2 Fig). Note that in our zero-inflated GLMs these effects are estimated for municipalities in which the binomial part of the models predicts that *R*. *pictipes* is likely to occur. The estimated odds of *R*. *pictipes* presence were lower in municipalities with higher diurnal temperatures or larger temperature amplitudes, and higher in rainier municipalities (S3 Table).

**Fig 4:**
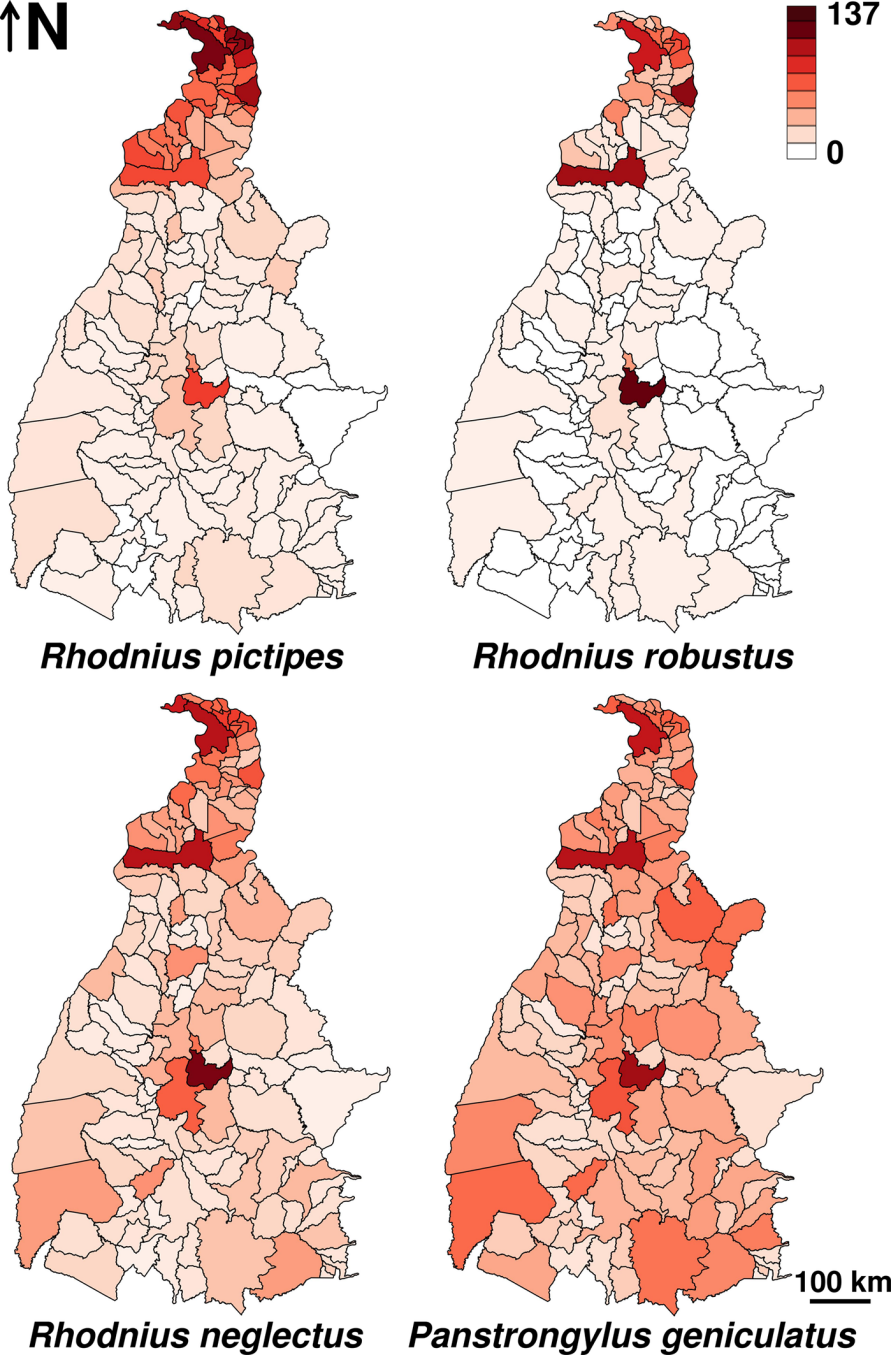
House invasion events (on a per-year basis) by four sylvatic triatomine species in Tocantins, Brazil, as predicted by generalized linear models. The maps show the limits of the 139 municipalities in the state of Tocantins. Darker shades of red indicate more invasion events; raw values were transformed to log_10_(*y*+1) to improve resolution at the lower end of the range. Model sets for each species are presented in Table 3 and S2 Table.

Per-year, model-averaged predictions for *R*. *pictipes* are presented in Fig 4. External validation with independent data collected in 2014–2016 revealed a good model-set performance (Table 4). Model-averaged, per-year predictions were lower than independent observations in the two northern municipalities consistently reporting the highest number of house-invasion events by *R*. *pictipes*–Araguatins (36.2 predicted *vs*. 113.7 per year observed in 2014–2016) and Tocantinópolis (25.2 *vs*. 119 per year in 2014–2016)–, as well as in Sítio Novo do Tocantins (26 *vs*. 93.7). At the other extreme, the models over-predicted moderately in Sampaio (18.9 predicted *vs*. 4 per year in 2014–2016) (see S3 Fig, S1 Data and S2 Table).

**Table 4:** Model-set performance metrics: Model predictions *vs*. independent observations over three years (2014–2016).

Species	Metric	Year*			
		2014	2015	2016	2014−2016
*Rhodnius pictipes*	Pearson’s *ρ*	0.778	0.791	0.722	0.794
	MBE	−0.997	−2.213	−3.184	−2.131
	MAE	2.912	3.997	4.740	3.747
	Within ±5 bugs	122 (87.8%)	121 (87.1%)	120 (86.3%)	121 (87.1%)
*Rhodnius robustus*	Pearson’s *ρ*	0.361	0.330	0.323	0.346
	MBE	0.691	0.332	0.216	0.413
	MAE	1.437	1.781	1.844	1.649
	Within ±5 bugs	132 (95.0%)	131 (94.2%)	129 (92.8%)	131 (94.2%)
*Rhodnius neglectus*	Pearson’s *ρ*	0.309	0.355	0.475	0.412
	MBE	0.197	−0.436	−0.098	−0.095
	MAE	2.315	2.611	2.357	2.169
	Within ±5 bugs	125 (89.9%)	122 (87.8%)	126 (90.7%)	124 (89.2%)
*Panstrongylus geniculatus*	Pearson’s *ρ*	0.346	0.463	0.432	0.464
	MBE	−0.428	−1.256	−1.385	−1.023
	MAE	2.565	3.204	3.298	2.737
	Within ±5 bugs	122 (87.8%)	124 (89.2%)	118 (84.9%)	123 (88.5%)

*Data for 2014–2016 became available after modeling was complete; the metrics in this Table compare the predictions of each species’ model set with the independent data for each year and with the (per-year) data of the three-year period (2014–2016). The performance metrics are: Pearson’s *ρ*, Pearson’s product moment correlation coefficient; MBE, mean bias error; MAE, mean absolute error; ‘Within ±5 bugs’, number (and percent) of municipalities where model-based predictions and independent observations differed by ±5 house-invading bugs or less

See S1 Data and S2 Table for details

### Rhodnius robustus

In 2010–2013, 653 *R*. *robustus*, many of them infected with *T*. *cruzi*, were caught in dwellings (97.9% found indoors) of 32 municipalities in Tocantins (Table 2, Fig 2). Female-bug invasion peaked in October-November, whereas male-bug invasion showed no monthly trends and was clearly less frequent; 74.9% (CI, 71.4–78.1%) of the 653 house-invading *R*. *robustus* were females.

Twelve *R*. *robustus* ZINB models had ΔAICc < 2.0 (Table 3). All but one of them included the *Rain* covariate on bug counts, and two were climate-only models differing only in the binomial part. Four of these models also included the regional *Amazon* covariate, and nine had landscape-scale covariates (Table 3). Neither null models nor simple regional- or landscape-scale models performed better than these 12 GLMs (S2 Table). The covariates that best explained the presence/absence of *R*. *robustus* in the binomial part of ZINB models were also related to temperature (S2 Table).

Model-averaged regression coefficients revealed clear negative effects of *Day* temperatures and *Rain* on house invasion by *R*. *robustus*. Although CIs were wider and often included zero, regional, landscape, and climate effects were (except for *Night* and *NDVI*) broadly comparable in size and sign to those estimated for *R*. *pictipes* (Fig 3; see also S3 and S4 Tables). The results of the *Poverty*-adjusted model set were qualitatively similar (see S2 Fig). Both *Day* and *ΔT* had strong negative effects on the odds of *R*. *robustus* presence (S4 Table).

Model-averaged, per-year predictions (Fig 4) were fairly accurate when compared to the independent 2014–2016 dataset (Table 4). The models, however, over-predicted house invasion by *R*. *robustus* in the two municipalities with the largest numbers of houses, Araguaína (~43,850 houses; 22.9 events predicted *vs*. 1 observed each year in 2014–2016) and Palmas (~68,680 houses; 77.8 predicted *vs*. none observed in 2014–2016). Under-prediction was moderate in Sítio Novo do Tocantins (7.1 *vs*. 26.7) and Tocantinópolis (28.6 *vs*. 47.3 per year observed in 2014–2016) (see S3 Fig, S1 Data and S2 Table).

### Rhodnius neglectus

House invasion by *T*. *cruzi*-infected *R*. *neglectus* was also common in Tocantins; from 2010 to 2013, 1118 *R*. *neglectus* were found in houses (92.4% indoors) of 93 municipalities (Table 2, Fig 2). Invasion appeared to be more frequent during the second half of the year, when female bias (overall, 60.0% females out of 1111 sexed specimens; CI, 57.1–62.9%) was also somewhat larger.

Table 3 presents the four top-ranking (ΔAICc < 2.0) *R*. *neglectus* negative binomial GLMs. The best-fit models included both climate (*Day* plus the quadratic *Rain* term) and landscape covariates; the regional covariate *Amazon* was present in one model in this subset (Table 3). We re-ran the top-ranking models (Table 3) leaving only linear *Rain* effects (i.e., without the quadratic term); all these alternative GLMs had ΔAICc > 3.9 (see S2 Table). Null and landscape-only models received no support from the data, while the best climate-only model had a ΔAICc > 2.9 (S2 Table). In line with expectations for a species with wide distribution over the Amazon–Cerrado transition, the regional-scale-only model received no support from the data (ΔAICc > 27.4; see S2 Table).

Model-averaged estimates of covariate effects on the number of house-invading *R*. *neglectus* are presented in Fig 3 and S5 Table. We found no evidence of regional effects. Similarly, although the frequency of house invasion was predicted to increase somewhat with the extent of municipal land at intermediate levels of disturbance and to decrease somewhat with more heavily-disturbed land, the CIs of landscape effects all encompassed zero. Alternative GLMs suggested a negative effect of *NDVI* (Fig 3, S5 Table). We found clear negative effects of higher diurnal temperatures and larger temperature amplitudes; the point estimate for *Night* was positive, although the lower limit of the CI was below zero in *HDI*-adjusted (but not in *Poverty*-adjusted) models (Fig 3, S2 Fig). Rainfall had a modest, non-linear effect, with house invasion by *R*. *neglectus* somewhat more frequent in municipalities close to the center of the rainfall gradient (where the standardized *Rain* covariate takes values near zero) and progressively less such events as rainfall came closer to extreme small and large values; the *Rain*^2^ effect CI derived from *Poverty*-adjusted models did not include zero (Fig 3, S5 Table, S2 Fig).

External validation suggested an acceptable average performance of the *R*. *neglectus* model set when confronted with independent data (Fig 4, Table 4). Over-prediction was moderate in the largest cities (Palmas, 32.3 predicted *vs*. 7.7 per year in 2014–2016; Araguaína, 18.4 *vs*. 8 per year), and the models under-predicted in the northeastern Tocantinópolis (7.5 *vs*. 27.7) and Babaçulândia (3.9 *vs*. 31.7) (see S3 Fig, S1 Data and S2 Table).

### Panstrongylus geniculatus

The catches of this widespread sylvatic species amounted to 2889 specimens in 2005–2013 (91.2% found indoors), with moderate natural infection rates (Table 2); 130 municipalities reported house invasion events by *P*. *geniculatus* across Tocantins (Fig 2). Both sexes were reported year-round, but males were more commonly caught: from 2732 sexed specimens, just 35.1% (CI, 33.3–36.9%) were females.

All *P*. *geniculatus* models with ΔAICc < 2.0 included the *Disturbed* landscape-scale covariate. None of these top-ranking models included the regional (*Amazon*) covariate, two included the quadratic *Rain* term, and all included temperature covariates *Day* or *ΔT* (Table 3). We re-fitted the two top-ranking models with rain effects after excluding the quadratic term, and found ΔAICc values of 1.67 and 2.60 (S2 Table). Again, joint models performed much better than null or single-category models–except that a landscape-only model with the *Disturbed* covariate had ΔAICc = 2.12 (S2 Table).

Model-averaged coefficients and their CIs were of overall smaller size for *P*. *geniculatus* than for *Rhodnius* spp. (Fig 3, S2 Fig). For all covariates except heavy landscape disturbance, which had a negative effect, the 95% CIs of slope coefficients included zero (Fig 3). Point estimates from *HDI*-adjusted models (Fig 3, S6 Table), as well as estimates and CIs from *Poverty*-adjusted models (S2 Fig), suggested, however, (i) that invasion was more frequent in municipalities with more land at intermediate disturbance levels and (ii) that warmer day temperatures and larger *ΔT* values were associated with moderate decreases of house invasion by *P*. *geniculatus*. Similarly, the effect of *Rain*^2^ was small but negative, again predicting more house invasion events in municipalities with annual rainfall closer to state-wide mean values (Fig 3, S2 Fig). We found no evidence of regional, NDVI, or night-temperature effects on house invasion by this species (Fig 3, S6 Table, S2 Fig).

Model-averaged predictions (Fig 4) matched fairly well the external validation dataset (Table 4). The models once again over-predicted in Palmas (23.1 predicted *vs*. 8 per year observed in 2014–2016) and Araguaína (18.1 *vs*. 8.3 per year); under-prediction was moderate, with differences ≤ −20 recorded in the northern Sítio Novo do Tocantins (5.8 predicted *vs*. 30.3 per year in 2014–2016) and the central-eastern Rio Sono (3 *vs*. 23.7) (S3 Fig, S1 Data and S2 Table).

## Discussion

This study shows that sylvatic triatomines frequently invade human dwellings across the transition between southeastern Amazonia and the Cerrado in Tocantins state, Brazil. Using a hypothesis-driven, multi-model inference approach coupled with external validation, we investigated what factors may underlie this invasive behavior. Our analyses clearly suggest that house invasion by four common sylvatic triatomine species is nonrandom across Tocantins municipalities, and indicate that some widely available environmental metrics may help predict the frequency of invasion at coarse geographic scales. These findings expand our understanding of a little-studied phenomenon of growing epidemiological relevance, and may help us develop a rational approach to Chagas disease risk prediction and stratification when house-colonizing vectors are absent [1,64,65].

### Drivers of house invasion

A first, general finding was that ‘joint’ models consistently outperformed single-scale or climate-only models (Tables 1 and 3 and S2 Table). Although two climate-only models were among the 12 top-ranking *R*. *robustus* models (Table 3), their Akaike weights added up to just 0.067 (S2 Table). This result provides a hint of the complexity of the drivers of house invasion by sylvatic triatomines, and suggests possible limitations of single-scale analyses–e.g., those based on models testing only regional, landscape, or climate effects.

We found support for the regional hypothesis of biome effects on the Amazonian *R*. *pictipes* and (with substantial uncertainty) *R*. *robustus*, but no regional effect was apparent for *R*. *neglectus* (Table 1, Fig 3, S2 Fig). Although *R*. *neglectus* is largely endemic to the Cerrado, some populations occur also in the moist forests of southeastern Amazonia (e.g., those described as ‘*R*. *milesi*’) and in other eco-regions adjacent to the Cerrado [38–40]. While continent-scale assessments reveal the broad association of *R*. *neglectus* with the savannahs of central Brazil [37,38], such association was not apparent along the complex Amazon–Cerrado transition in our study region (Figs 1–4, S5 Table). As expected, we found no measurable biome effect on *P*. *geniculatus* (Table 1, Fig 3, S6 Table, S2 Fig).

Our evaluation of the landscape-disturbance hypothesis (Table 1) revealed two distinct patterns of response. The Amazonian, forest-dwelling *R*. *pictipes* and (to a lesser extent) *R*. *robustus* appeared to respond linearly, with more invasion events in municipalities with better-preserved landscapes and a trend towards less invasion in municipalities with more heavily-disturbed land; uncertainty about effect estimates was however substantial, particularly for *R*. *robustus* (Fig 3, S2 Fig). For *R*. *neglectus* and *P*. *geniculatus*, our models suggest that invasion events may be more frequent in municipalities with more land at intermediate levels of disturbance, and less frequent in municipalities with more heavily-disturbed landscapes (Fig 3, S2 Fig). We therefore found no evidence of a negative association between well-preserved land and house invasion (Table 1, Fig 3, S2 Fig). Instead, our results suggest that the loss of suitable habitat (and perhaps hosts) at the landscape scale is detrimental for sylvatic triatomine populations–which, even if possibly present, may be rarer (with fewer and/or lower-density foci) in heavily-disturbed than in better-preserved environments [13,15,38,40,52,66]. The possible effects of intermediate disturbance levels on *R*. *neglectus* and *P*. *geniculatus* might indicate that open eco-region species (such as *R*. *neglectus*) and broad eco-region generalists (such as *P*. *geniculatus*) benefit from mild disturbance in a way that forest-specialist species such as *R*. *pictipes* or *R*. *robustus* do not [13,67]. In interpreting these contrasting findings, however, one should keep in mind that the results of ZINB models used for Amazonian *Rhodnius* spp. refer only to the municipalities where these bugs were predicted to occur. As with other parts of our coarse-scale analysis, we also stress that these quantitative results are valuable in that they hint at specific, landscape-scale hypotheses that can be tested with landscape-scale research [52,53,66]. For example, previous landscape-scale analyses have suggested relatively weak disturbance effects on palm infestation by *Rhodnius* spp., with somewhat higher estimates in rural than in either forested or urban sites [38,40,66]. In rural Panama, *R*. *pallescens* foci are apparently denser, and the bugs more often infected with *T*. *cruzi*, in palms of more disturbed *vs*. better preserved landscapes [52,53]. House invasion by *Triatoma vitticeps*, a species associated with stone-ground habitats, appears to be more frequent in intermediate-disturbance sites where preserved patches of Atlantic forest [13] or Campos Rupestres savannahs [68] persist on stony hill-slopes. Similarly, spatially-explicit models suggest that wild bug populations from sylvatic/agricultural mixed landscapes can contribute substantially (albeit probably less than peridomestic foci) to house invasion by adult *T*. *dimidiata* in rural Yucatán, Mexico [10]. A few studies indicate, finally, that some arboreal triatomine species, such as *T*. *tibiamaculata* or *P*. *megistus*, manage to survive in urban forest remnants [15,40,69,70].

We also examined the more proximate hypothesis that ambient temperature may impose physiological constraints on triatomine survival and population growth or affect dispersive flight performance [54–58] (Table 1). We found clear evidence that house invasion events are rarer in hotter-day municipalities of Tocantins, with stronger effects on *Rhodnius* spp. than on *P*. *geniculatus* (Fig 3, S2 Fig). The evidence for a positive effect of warmer nights was much less consistent, perhaps because of the smaller variation in nocturnal (SD = 0.44°C) than diurnal (SD = 1.38°C) mean temperatures (Fig 3, S2 Fig and S1 Table). Temperature amplitude (*ΔT*) was also associated with less house invasion events, most likely through the dominant effect of high diurnal temperatures (Fig 3, S2 Fig). We again suggest that these results are best viewed as hints of specific hypotheses about the effects of temperature on the bugs’ fitness (and dispersal) that can be tested with targeted research designs. Lastly, we considered the possibility that the heavy, seasonal rains typical of Tocantins might hinder dispersive flight by sylvatic triatomines [57,58] (Table 1). We found evidence of rainfall effects on house invasion by all four focal species–with, again, two different response patterns. The effect was linear and negative for *R*. *pictipes* and *R*. *robustus*, suggesting that heavy rain may interfere with flight in the cooler-day (and, for *R*. *pictipes*, overall somewhat rainier) municipalities where these Amazonian bugs occur (Fig 3, S2 Fig, S3 and S4 Tables). This is consistent with results from other Amazon sub-regions, where more dispersing sylvatic triatomines are typically caught at light traps during the drier season [71,72]. Rain effects were also negative, yet non-linear, for *R*. *neglectus* and *P*. *geniculatus*, with a quadratic term providing the best fit to the data (S2 Table). This suggests that populations of these two species may be rarer, or perhaps less dense, in municipalities towards the drier end of the rainfall range in Tocantins; at rainier sites, heavy rainfall may hinder these bugs’ dispersive flight, albeit to a lesser extent than for Amazonian *Rhodnius* spp. (Fig 3, S2 Fig and S3–S6 Tables) [57,58,71,72].

### Potential applications

Although largely exploratory, coarse-grained, and based on imperfect data (see **Caveats** below), our cross-scale model sets performed fairly well at predicting ‘future’ invasion events in the independent validation dataset (Figs 2 and 4 and S3 Fig). Together with the very poor relative performance of null models (S2 Table), this suggests that the models successfully captured important drivers of house invasion by sylvatic triatomines. In practical terms, these results may be seen as a step towards Chagas disease risk stratification and mapping in the absence of house-colonizing vectors. This could be useful across most of the Americas, where sylvatic triatomines are widespread and often invade human dwellings [1,64,65]; the broad availability of environmental and socio-economic datasets such as those we used here should facilitate this application. There were, however, some mismatches between model predictions and the validation dataset. Substantial under-prediction might indicate poor model performance, but this was the case in only a few municipalities (S2 Table, S3 Fig). That most models over-predicted in the two municipalities with the largest urban centers reflects our strategy of adjusting for the *House* confounder. *House* coefficients were positive across species, while *HDI* had either negative (for *P*. *geniculatus*) or non-measurable effects (S3–S6 Tables). On the other hand, some municipalities produced moderately fewer records than predicted by their coarse-scale environmental and socio-demographic characteristics (S2 Table, S3 Fig). If consistent across vector species, such a mismatch might signal under-performance of local vector surveillance. This interpretation, however, is made with great caution, because our models offer just a rough approximation to an obviously complex phenomenon.

### Generality of the approach

Taken as a whole, our results show how different climatic and landscape-level factors can have distinct effects on the invasive behavior of different triatomine species. Both the relative importance of covariates and the model-averaged estimates of their coefficients suggest, for example, (i) that *Rhodnius* spp. are overall more sensitive to climate effects than the widespread *P*. *geniculatus*, (ii) that *R*. *neglectus* and *P*. *geniculatus* may adapt better than the forest-dwelling *R*. *pictipes* and *R*. *robustus* to intermediate levels of anthropogenic landscape disturbance, or (iii) that excess rainfall may hinder flight by Amazonian *Rhodnius* species more than it does by their Cerrado close relative, *R*. *neglectus*. This indicates that common sources of environmental stress can trigger idiosyncratic responses in different triatomine taxa, and therefore that the results of this and similar studies may not apply to bug species that were not investigated. We nonetheless believe that the general approach we have described can be useful under a wide range of circumstances; it requires (i) a clear research question on a defined set of vector species, (ii) a suite of plausible *a priori* hypotheses represented by properly specified competing models, (iii) data to fit the models plus sound criteria to evaluate model performance, (iv) inference based on each species’ full model set, and (v) independent data for external model validation [10,49,50,62,73,74]. Our application of such a robust approach to a large dataset is, we believe, the most salient strength of this report–which comes, however, with some caveats.

### Caveats

It should first be noted that our vector surveillance data likely account for just a small fraction of all the house-invasion events that occurred in the study area and period. Hence, such events are probably more common and widespread than we report here. This also suggests that part of the among-municipality variation in house invasion events left unexplained by our models may be due to variation in the performance of municipal vector surveillance systems. Our use of multi-year data in model fitting and external validation likely mitigated this potential problem, but cannot be expected to fully solve it; the results should therefore be interpreted with caution. This inherent limitation of the data is the main reason why we chose to present general validation metrics (Pearson’s *ρ*, MBE, and MAE) instead of emphasizing detailed, municipality-by-municipality quantitative comparisons.

Second, our use of municipality- and time-aggregated house-invasion data means that we disregard within-municipality and between-year variations. While the latter seems reasonable for the time-frame of our analyses, variation among sites (and houses) within municipalities is clearly to be expected [10,11,57,75]. We were unable to analyze house invasion events at the site (let alone house) level because the geographic coordinates of most of the (many) small, remote rural sites in the dataset were impossible to retrieve; furthermore, the subset of sites for which coordinates were available probably contains a biased sample of all sites–larger, less isolated localities are more likely to be in that subset. We therefore make no claims as to what might drive among-site variation within municipalities. We emphasize, at the same time, that current vector control-surveillance systems operate at the municipality level in Tocantins and elsewhere in Brazil, so that overall measures of vector-borne disease risk represent operationally valuable information. Our analyses illustrate how such information can be recovered from entomological routine surveillance data–which are often generated and stockpiled with substantial effort but whose potential is seldom realized.

Finally, we investigated just a few covariates from the vast set of all possible drivers of triatomine invasive behavior. We based *a priori* covariate choice on our knowledge about the biogeography, ecology, behavior, and physiology (with special attention to dispersive flight) of the four focal species. This allowed us to formulate a set of clear hypotheses and to pre-specify their predictions (see Methods and Table 1). Apart from being biologically informed, which eased the interpretation of results, this covariate selection allowed us to develop a relatively parsimonious, yet thorough, data-analytical strategy (S2 Table).

### Conclusions and outlook

This report shows that house invasion by sylvatic triatomine bugs is far more widespread and frequent than usually perceived. Our analyses suggest, in addition, that some readily available environmental predictors may help assess the coarse-scale risk of Chagas disease transmission by sylvatic vectors. Although this stands out as a priority in areas (such as eastern Amazonia) where house-invading vectors have repeatedly been implicated in acute Chagas disease outbreaks, enhanced risk assessment should be helpful wherever native triatomines can transmit *T*. *cruzi* to people [64,65,76]. Our analyses also identified several specific hypotheses for finer-scale research. For example, survival and population growth of sylvatic triatomines may be limited in hotter-day regions or in heavily disturbed landscapes, whereas some species may benefit from intermediate levels of anthropogenic disturbance. Our study illustrates, in sum, how joint analyses of vector surveillance records and eco-regional, landscape, and climate data may help advance Chagas disease risk prediction and stratification when house-colonizing vectors are absent.

## Supporting information

S1 Data Raw data. Municipality names, numbers of house-invading bugs, regional covariate (*Amazon*, %), landscape covariates (*Preserved*, *Intermediate*, *Disturbed*, %; *NDVI*, no units), climate covariates: temperature (*Day*, *Night*, *ΔT*, in °C) and rainfall (*Rain*, in mm), confounders (inhabited *Houses*, Human Development Index [*HDI*], % *Poverty*), and external validation data (house-invasion events in 2014 to 2016).
(XLSX)

S1 Fig Mapped values of covariates and confounders. The maps show the limits of the 139 municipalities of Tocantins; darker shades indicate larger values. See also S1 Data.
(PDF)

S2 Fig Model-averaged estimates of covariate effects on house invasion by four sylvatic triatomine species: *Poverty*-adjusted model sets. Circles, regional-scale covariate (*Amazon*); diamonds, landscape-scale covariates (*Preserved*, *Intermediate*, *Disturbed*, *NDVI*); squares, climate covariates (*Day*, *Night*, *ΔT*, *Rain*; *Rain*2, quadratic *Rain* term; Rain*, estimate and confidence interval (CI) from the top-ranking model). Effects are considered different from zero (black symbols) when the 95% CIs do not cross the horizontal line at zero.
(PDF)

S3 Fig External model validation. Frequency histograms of the differences between model-averaged predictions and independent 2014–2016 data for each species. Inset maps show model-predicted and independent validation data (on a per-year basis) in each municipality (scale as in Figs 2 and 4); see also S1 Data. ZINB, zero-inflated negative binomial and NB, negative binomial generalized linear models.
(PDF)

S1 Table Covariates and confounders. Categories, units, names, and summary statistics of covariates and confounders over the 139 municipalities of Tocantins, Brazil.
(PDF)

S2 Table The complete model sets. For each species, we present all models, grouped by category, as well as their structure, number of parameters (K), and performance metrics including AICc, ΔAICc, Akaike weights (individual and cumulative [CumWt]), and log-likelihood (LL). Model-averaged predictions and independent validation data (2014–2016, on a per-year basis) are also shown for each municipality.
(XLSX)

S3 Table *Rhodnius pictipes* zero-inflated negative binomial generalized linear models. Model-averaged coefficients, unconditional standard errors (SE), and 95% confidence interval limits (CI_lower_, CI_upper_) from 139 models (123 without convergence issues) fitted for this species.
(PDF)

S4 Table *Rhodnius robustus* zero-inflated negative binomial generalized linear models. Model-averaged coefficients, unconditional standard errors (SE), and 95% confidence interval limits (CI_lower_, CI_upper_) from 219 models fitted for this species.
(PDF)

S5 Table *Rhodnius neglectus* negative binomial generalized linear models. Model-averaged coefficients, unconditional standard errors (SE), and 95% confidence interval limits (CI_lower_, CI_upper_) from 87 models fitted for this species.
(PDF)

S6 Table *Panstrongylus geniculatus* negative binomial generalized linear models. Model-averaged coefficients, unconditional standard errors (SE), and 95% confidence interval limits (CI_lower_, CI_upper_) from 89 models fitted for this species.
(PDF)
