# Supplementary material for: Drivers of house invasion by sylvatic Chagas disease vectors in the Amazon-Cerrado transition: A multi-year, state-wide assessment of municipality-aggregated surveillance data
Source: PLoS Negl Trop Dis. 2017 Nov 16;11(11):e0006035. doi: 10.1371/journal.pntd.0006035 (PMC5689836; doi:10.1371/journal.pntd.0006035)
Supplement: S1 Fig — The maps show the limits of the 139 municipalities of Tocantins; darker shades indicate larger values. See also S1 Data. (PDF) [file pntd.0006035.s002.pdf]

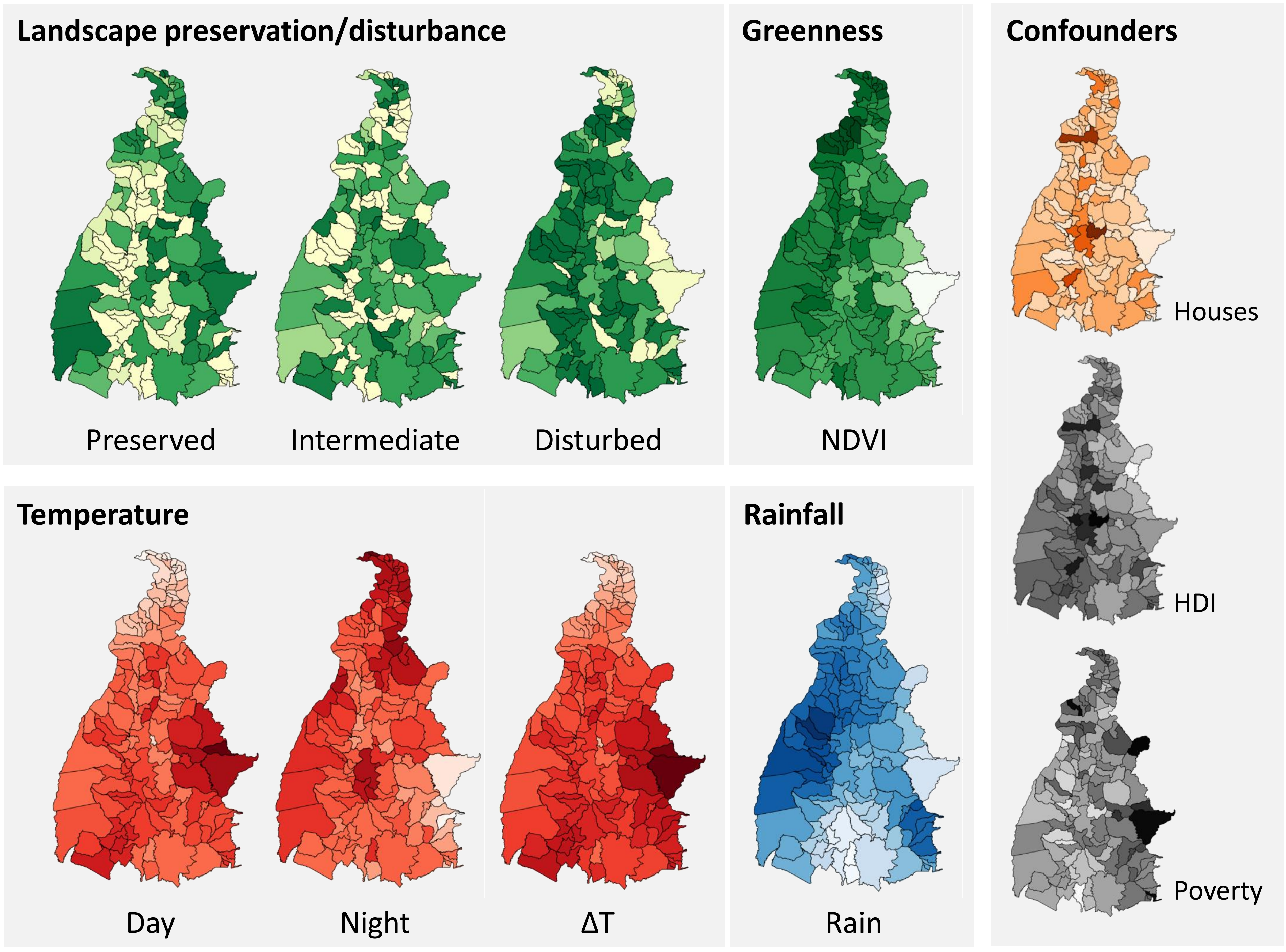
Landscape preservation/disturbance
Preserved
Intermediate
Disturbed
Greenness
NDVI
Confounders
Houses
HDI
Poverty
Temperature
Day
Night
ΔT
Rainfall
Rain
