# Supplementary material for: Drivers of house invasion by sylvatic Chagas disease vectors in the Amazon-Cerrado transition: A multi-year, state-wide assessment of municipality-aggregated surveillance data
Source: PLoS Negl Trop Dis. 2017 Nov 16;11(11):e0006035. doi: 10.1371/journal.pntd.0006035 (PMC5689836; doi:10.1371/journal.pntd.0006035)
Supplement: S2 Fig — Circles, regional-scale covariate (Amazon); diamonds, landscape-scale covariates (Preserved, Intermediate, Disturbed, NDVI); squares, climate covariates (Day, Night, ΔT, Rain; Rain2, quadratic Rain term; Rain*, estimate and confidence interval (CI) from the top-ranking model). Effects are considered different from zero (black symbols) when the 95% CIs do not cross the horizontal line at zero. (PDF) [file pntd.0006035.s003.pdf]

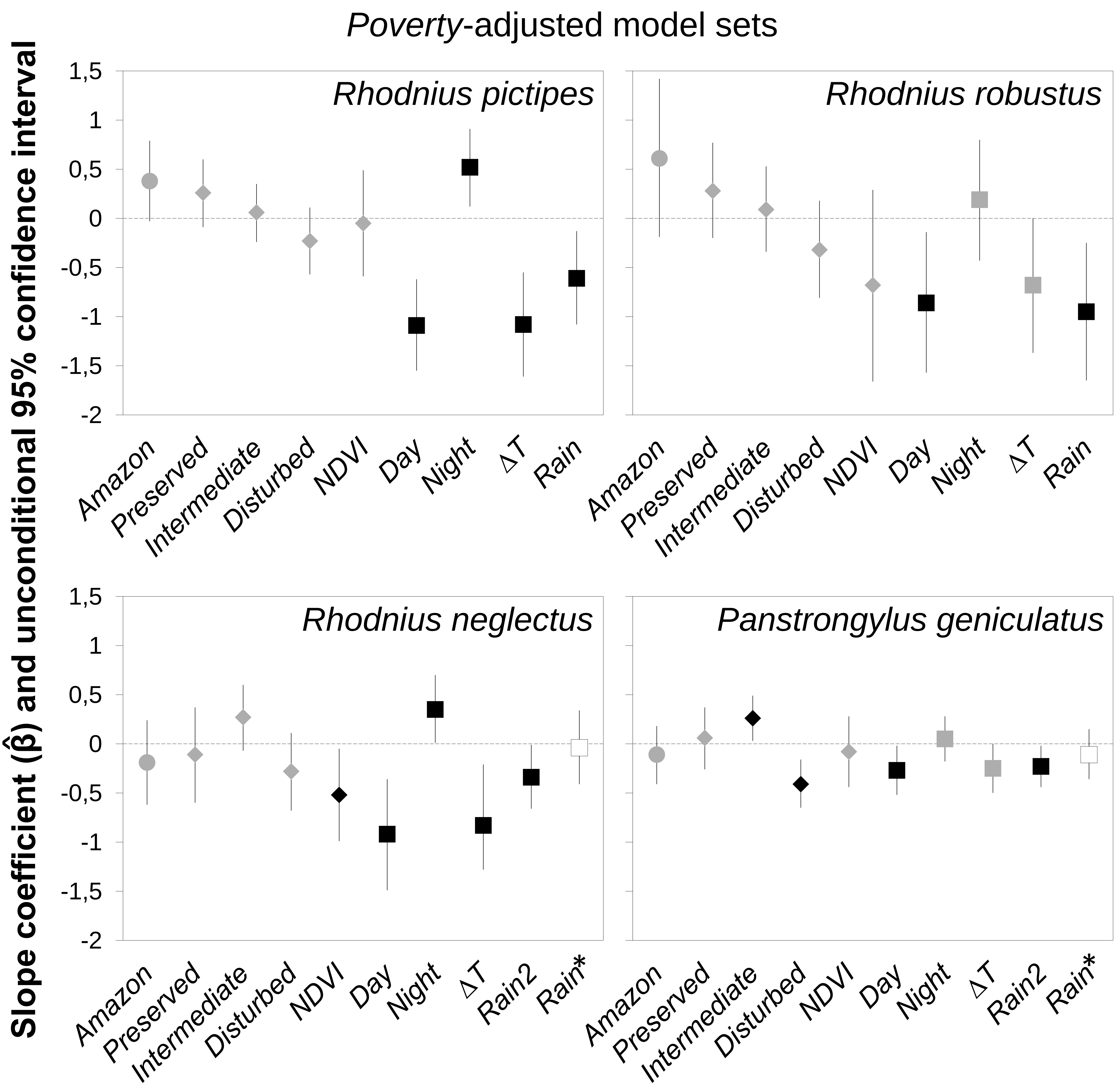
Poverty-adjusted model sets
Slope coefficient (β̂) and unconditional 95% confidence interval
Rhodnius pictipes
Rhodnius robustus
Rhodnius neglectus
Panstrongylus geniculatus
1,5
1
0,5
0
-0,5
-1
-1,5
-2
Amazon
Preserved
Intermediate
Disturbed
NDVI
Day
Night
ΔT
Rain
Rain2
Rain*
