# Supplementary figures and images for: Drivers of house invasion by sylvatic Chagas disease vectors in the Amazon-Cerrado transition: A multi-year, state-wide assessment of municipality-aggregated surveillance data
Source: PLoS Negl Trop Dis. 2017 Nov 16;11(11):e0006035. doi: 10.1371/journal.pntd.0006035 (PMC5689836; doi:10.1371/journal.pntd.0006035)

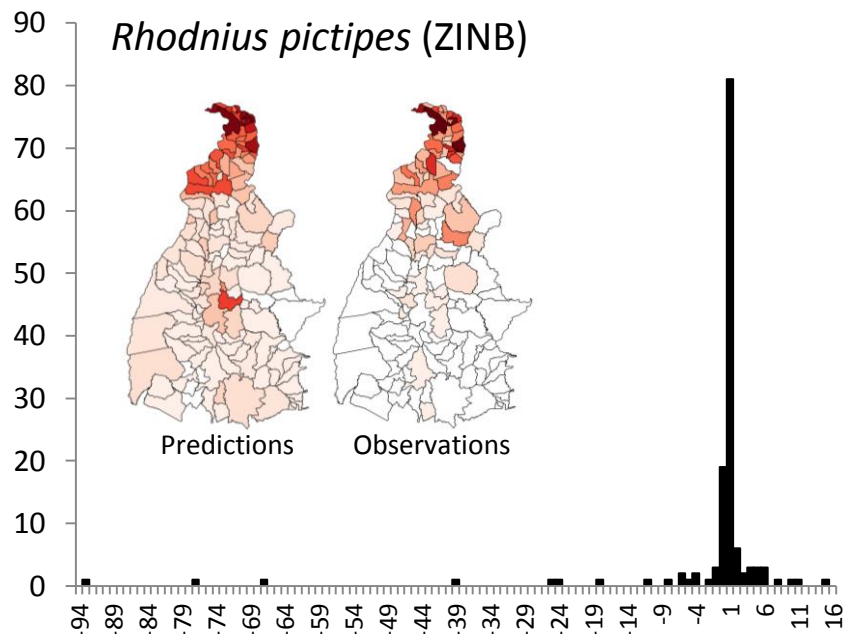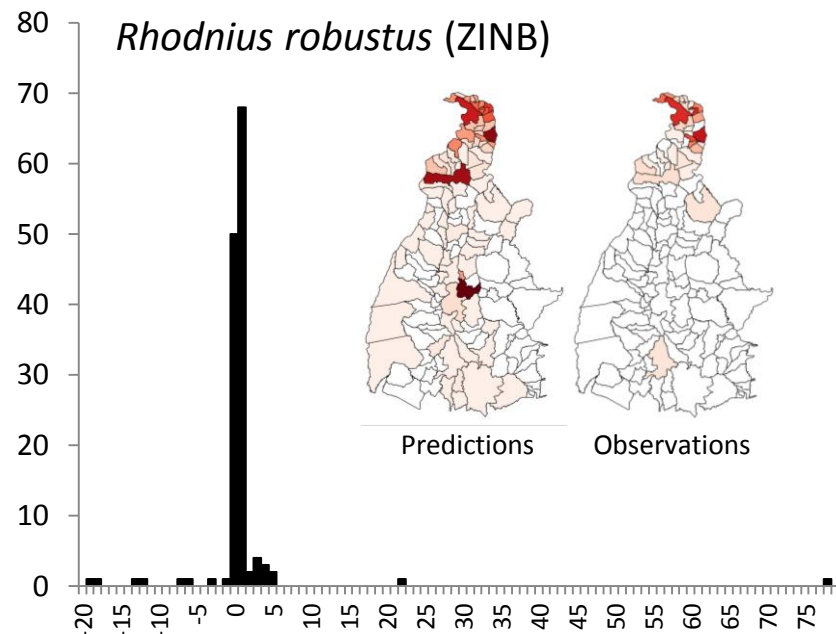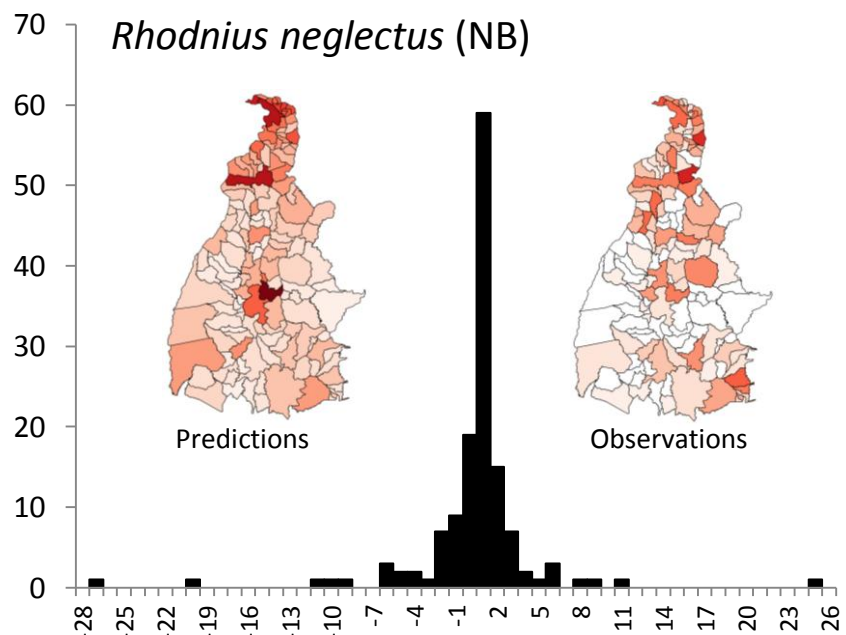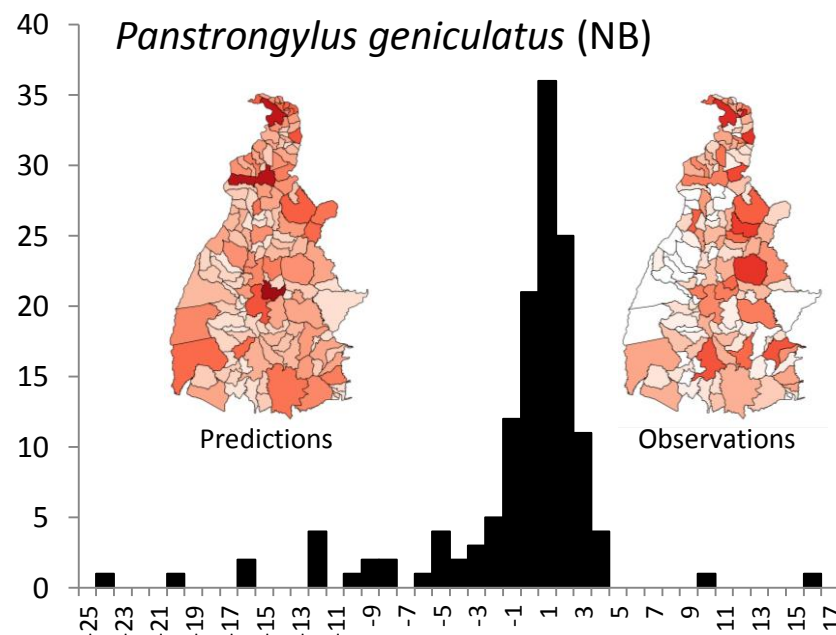

Predictions (model-averaged, per-year) – Observations (independent dataset, 2014–2016)

Supplement: S3 Fig — Frequency histograms of the differences between model-averaged predictions and independent 2014–2016 data for each species. Inset maps show model-predicted and independent validation data (on a per-year basis) in each municipality (scale as in Figs 2 and 4); see also S1 Data. ZINB, zero-inflated negative binomial and NB, negative binomial generalized linear models. (PDF) [file pntd.0006035.s004.pdf]
