# Supplementary material for: Drivers of house invasion by sylvatic Chagas disease vectors in the Amazon-Cerrado transition: A multi-year, state-wide assessment of municipality-aggregated surveillance data
Source: PLoS Negl Trop Dis. 2017 Nov 16;11(11):e0006035. doi: 10.1371/journal.pntd.0006035 (PMC5689836; doi:10.1371/journal.pntd.0006035)
Supplement: S1 Table — Categories, units, names, and summary statistics of covariates and confounders over the 139 municipalities of Tocantins, Brazil. (PDF) [file pntd.0006035.s005.pdf]

S1 Table. Covariates and confounders. Categories, units, names, and summary statistics of covariates and confounders over the 139 municipalities of Tocantins, Brazil.

Category	Covariate (units)	Name	Mean	SD	Range	
Regional	Amazon (%)	*Amazon*	41.37	40.20	0.00	100.00
Landscape	Well preserved land (%)	*Preserved*	20.69	28.58	0.00	95.00
	Land at intermediate disturbance (%)	*Intermediate*	22.67	28.02	0.00	95.30
	Heavily disturbed land (%)	*Disturbed*	50.86	37.20	0.03	96.20
	NDVI (unitless ratio; −1.0 to 1.0)	*NDVI*	0.63	0.04	0.45	0.69
Climate	Day temperature (°C)	*Day*	32.51	1.38	29.19	36.06
	Night temperature (°C)	*Night*	22.27	0.44	20.80	23.30
	Temperature amplitude (°C)	ΔT	10.23	1.64	6.02	14.25
	Rainfall (mm)	*Rain*	4103	357	3259	4776
Confounders	Houses (number)	*House*	2866	7177	319	68,679
	Human Development Index (0 to 1)	*HDI*	0.64	0.05	0.50	0.79
	Poverty (%)	*Poverty*	45.11	10.71	25.71	84.00

SD, standard deviation; NDVI, Normalized Difference Vegetation Index; Temperature amplitude, difference between day and night mean temperatures
