# Supplementary material for: Drivers of house invasion by sylvatic Chagas disease vectors in the Amazon-Cerrado transition: A multi-year, state-wide assessment of municipality-aggregated surveillance data
Source: PLoS Negl Trop Dis. 2017 Nov 16;11(11):e0006035. doi: 10.1371/journal.pntd.0006035 (PMC5689836; doi:10.1371/journal.pntd.0006035)
Supplement: S3 Table — Model-averaged coefficients, unconditional standard errors (SE), and 95% confidence interval limits (CIlower, CIupper) from 139 models (123 without convergence issues) fitted for this species. (PDF) [file pntd.0006035.s007.pdf]

S3 Table. ***Rhodnius pictipes*** **zero-inflated negative binomial generalized linear models.** Model-averaged coefficients, unconditional standard errors (SE), and 95% confidence interval limits (CI_{lower}, CI_{upper}) from 139 models (123 without convergence issues) fitted for this species.

Category	Covariate	Estimate	SE	CI_{lower}	CI_{upper}
Negative binomial (count) part – modeling the number of house invasion events					
Intercept	-	−3.18	1.54	−6.20	−0.17
Regional-scale	*Amazon*	0.49	0.21	0.08	0.89
Landscape-scale	*Preserved*	0.24	0.16	−0.08	0.55
	Intermediate	0.02	0.14	−0.24	0.29
	Disturbed	−0.18	0.17	−0.51	0.15
	NDVI	0.17	0.27	−0.36	0.71
Climate	*Day*	−0.90	0.25	−1.39	−0.40
	Night	0.26	0.23	−0.20	0.72
	ΔT	−0.91	0.27	−1.45	−0.38
	Rain	−0.67	0.22	−1.10	−0.23
Confounders	*House*	0.73	0.21	0.32	1.13
	HDI	−0.35	0.19	−0.73	0.03
Binomial (zero inflation) part – modeling the odds of species *absence*					
Intercept	-*	−0.34	0.34	−1.01	0.32
Regional-scale	*Amazon**	−0.23	0.40	−1.02	0.56
Climate	*Day*	1.45	0.41	0.64	2.26
	ΔT	1.90	0.44	1.04	2.77
	Rain	−0.91	0.38	−1.66	−0.17

Since all covariates were standardized, effect estimates measure the expected increase or decrease in house-invasion event counts for each one-standard deviation increase (from the zero mean) in the covariate value (see S1 Table)

NDVI, the Normalized Difference Vegetation Index, was used as a single-figure alternative to landscape-scale disturbance classes; *ΔT*, temperature amplitude, or the difference between diurnal and nocturnal mean temperatures

*Excluding 16 models (Akaike weights ≤ 0.005) with convergence issues in the binomial part
