# Supplementary material for: Drivers of house invasion by sylvatic Chagas disease vectors in the Amazon-Cerrado transition: A multi-year, state-wide assessment of municipality-aggregated surveillance data
Source: PLoS Negl Trop Dis. 2017 Nov 16;11(11):e0006035. doi: 10.1371/journal.pntd.0006035 (PMC5689836; doi:10.1371/journal.pntd.0006035)
Supplement: S4 Table — Model-averaged coefficients, unconditional standard errors (SE), and 95% confidence interval limits (CIlower, CIupper) from 219 models fitted for this species. (PDF) [file pntd.0006035.s008.pdf]

S4 Table. *Rhodnius robustus* zero-inflated negative binomial generalized linear models. Model-averaged coefficients, unconditional standard errors (SE), and 95% confidence interval limits (CI_{lower}, CI_{upper}) from 219 models fitted for this species.

Category	Covariate	Estimate	SE	CI_{lower}	CI_{upper}
Negative binomial (count) part – modeling the number of house invasion events					
Intercept	-	−7.85	3.04	−13.81	−1.89
Regional-scale	*Amazon*	0.53	0.43	−0.32	1.38
Landscape-scale	*Preserved*	0.23	0.27	−0.31	0.76
	Intermediate	0.18	0.24	−0.29	0.65
	Disturbed	−0.36	0.27	−0.89	0.16
	NDVI	−0.71	0.49	−1.67	0.25
Climate	*Day*	−1.37	0.60	−2.55	−0.18
	Night	−0.08	0.43	−0.92	0.76
	ΔT	−1.00	0.61	−2.20	0.20
	Rain	−0.87	0.40	−1.66	−0.08
Confounders	*House*	1.07	0.44	0.21	1.94
	HDI	0.34	0.60	−0.84	1.52
Binomial (zero inflation) part – modeling the odds of species *absence*					
Intercept	-	1.52	0.74	0.07	2.97
Regional-scale	*Amazon*	−0.03	0.66	−1.32	1.27
Climate	*Day*	3.27	1.00	1.30	5.23
	ΔT	3.10	0.89	1.36	4.85
	Rain	−0.02	0.70	−1.39	1.35

Since all covariates were standardized, effect estimates measure the expected increase or decrease in house-invasion event counts for each one-standard deviation increase (from the zero mean) in the covariate value (see S1 Table)

NDVI, the Normalized Difference Vegetation Index, was used as a single-figure alternative to landscape-scale disturbance classes; *ΔT*, temperature amplitude, or the difference between diurnal and nocturnal mean temperatures
