# Supplementary material for: Drivers of house invasion by sylvatic Chagas disease vectors in the Amazon-Cerrado transition: A multi-year, state-wide assessment of municipality-aggregated surveillance data
Source: PLoS Negl Trop Dis. 2017 Nov 16;11(11):e0006035. doi: 10.1371/journal.pntd.0006035 (PMC5689836; doi:10.1371/journal.pntd.0006035)
Supplement: S5 Table — Model-averaged coefficients, unconditional standard errors (SE), and 95% confidence interval limits (CIlower, CIupper) from 87 models fitted for this species. (PDF) [file pntd.0006035.s009.pdf]

S5 Table. *Rhodnius neglectus* negative binomial generalized linear models. Model-averaged coefficients, unconditional standard errors (SE), and 95% confidence interval limits (CI_{lower}, CI_{upper}) from 87 models fitted for this species.

Category	Covariate	Estimate	SE	CI_{lower}	CI_{upper}
Intercept	-	−3.73	1.77	−7.19	−0.26
Regional-scale	*Amazon*	−0.18	0.22	−0.62	0.26
Landscape-scale	*Preserved*	−0.10	0.24	−0.58	0.37
	Intermediate	0.24	0.17	−0.09	0.58
	Disturbed	−0.27	0.21	−0.67	0.14
	NDVI	−0.50	0.23	−0.96	−0.04
Climate	*Day*	−0.89	0.28	−1.44	−0.34
	Night	0.28	0.19	−0.08	0.65
	ΔT	−0.81	0.24	−1.27	−0.35
	$Rain^2$	−0.34	0.17	−0.66	−0.02
	*Rain**	−0.05	0.17	−0.43	0.34
Confounders	*House*	0.74	0.24	0.28	1.20
	HDI	−0.08	0.21	−0.49	0.33

Since all covariates were standardized, effect estimates measure the expected increase or decrease in house-invasion event counts for each one-standard deviation increase (from the zero mean) in the covariate value (see S1 Table)

NDVI, the Normalized Difference Vegetation Index, was used as a single-figure alternative to landscape-scale disturbance classes; ΔT, temperature amplitude, or the difference between diurnal and nocturnal mean temperatures

*Estimate, SE, and CI from the top-ranking model (see Table 3 and S2 Table)
