# Supplementary material for: Drivers of house invasion by sylvatic Chagas disease vectors in the Amazon-Cerrado transition: A multi-year, state-wide assessment of municipality-aggregated surveillance data
Source: PLoS Negl Trop Dis. 2017 Nov 16;11(11):e0006035. doi: 10.1371/journal.pntd.0006035 (PMC5689836; doi:10.1371/journal.pntd.0006035)
Supplement: S6 Table — Model-averaged coefficients, unconditional standard errors (SE), and 95% confidence interval limits (CIlower, CIupper) from 89 models fitted for this species. (PDF) [file pntd.0006035.s010.pdf]

S6 Table. ***Panstrongylus geniculatus*** **negative binomial generalized linear models.**

Model-averaged coefficients, unconditional standard errors (SE), and 95% confidence interval limits (CI_{lower}, CI_{upper}) from 89 models fitted for this species.

Category	Covariate	Estimate	SE	CI_{lower}	CI_{upper}
Intercept	-	−4.46	1.13	−6.67	−2.26
Regional-scale	*Amazon*	−0.01	0.14	−0.28	0.26
Landscape-scale	*Preserved*	0.07	0.15	−0.21	0.36
	Intermediate	0.15	0.10	−0.05	0.35
	Disturbed	−0.27	0.11	−0.49	−0.04
	NDVI	0.06	0.14	−0.22	0.35
Climate	*Day*	−0.20	0.11	−0.43	0.02
	Night	−0.02	0.11	−0.24	0.20
	ΔT	−0.18	0.11	−0.40	0.05
	$Rain^2$	−0.18	0.10	−0.38	0.01
	*Rain**	−0.07	0.11	−0.30	0.16
Confounders	*House*	0.98	0.15	0.69	1.28
	HDI	−0.43	0.14	−0.70	−0.16

Since all covariates were standardized, effect estimates measure the expected increase or decrease in house-invasion event counts for each one-standard deviation increase (from the zero mean) in the covariate value (see S1 Table)

NDVI, the Normalized Difference Vegetation Index, was used as a single-figure alternative to landscape-scale disturbance classes; ΔT, temperature amplitude, or the difference between diurnal and nocturnal mean temperatures

*Estimate, SE, and CI from the top-ranking model (see Table 3 and S2 Table)
